# The Kidney Score Platform for Patient and Clinician Awareness, Communication, and Management of Kidney Disease: Protocol for a Mixed Methods Study

**DOI:** 10.2196/22024

**Published:** 2020-10-19

**Authors:** Delphine S Tuot, Susan T Crowley, Lois A Katz, Joseph Leung, Delly K Alcantara-Cadillo, Christopher Ruser, Elizabeth Talbot-Montgomery, Joseph A Vassalotti

**Affiliations:** 1 University of California, San Francisco San Francisco, CA United States; 2 Veterans Affairs Connecticut Healthcare System West Haven, CT United States; 3 Yale University School of Medicine New Haven, CT United States; 4 Veterans Affairs New York Harbor Healthcare System New York, NY United States; 5 New York University Grossman School of Medicine New York, NY United States; 6 National Kidney Foundation New York, NY United States; 7 Icahn School of Medicine at Mount Sinai New York, NY United States

**Keywords:** chronic kidney disease, CKD, awareness, implementation science, behavioral change wheel, RE-AIM

## Abstract

**Background:**

Patient awareness, clinician detection, and management of chronic kidney disease remain suboptimal, despite clinical practice guidelines and diverse education programs.

**Objective:**

This protocol describes a study to develop and investigate the impact of the National Kidney Foundation Kidney Score Platform on chronic kidney disease awareness, communication, and management, by leveraging the Behavior Change Wheel, an implementation science framework that helps identify behavioral intervention targets and functions that address barriers to behavior change.

**Methods:**

We interviewed 20 patients with chronic kidney disease and 11 clinicians to identify patient and clinician behaviors suitable for intervention and barriers to behavior change (eg, limited awareness of chronic kidney disease clinical practice guidelines within primary care settings, limited data analytics to highlight chronic kidney disease care gaps, asymptomatic nature of chronic kidney disease in conjunction with patient reliance on primary care clinicians to determine risk and order kidney testing). Leveraging the Behavior Change Wheel, the Kidney Score Platform was developed with a patient-facing online Risk Calculator and a clinician-facing Clinical Practice Toolkit. The Risk Calculator utilizes risk predictive analytics to provide interactive health information tailored to an individual’s chronic kidney disease risk and health status. The Clinical Practice Toolkit assists clinicians in discussing chronic kidney disease with individuals at risk for and with kidney disease and in managing their patient population with chronic kidney disease. The Kidney Score Platform will be tested in 2 Veterans Affairs primary health care settings using a pre–post study design. Outcomes will include changes in patient self-efficacy for chronic kidney disease management (primary outcome), quality of communication with clinicians about chronic kidney disease, and practitioners’ knowledge of chronic kidney disease guidelines. Process outcomes will identify usability and adoption of different elements of the Kidney Score Platform using qualitative and quantitative methods.

**Results:**

As of September 2020, usability studies are underway with veterans and clinicians to refine the patient-facing components of the Kidney Score Platform before study initiation. Results and subsequent changes to the Kidney Score Platform will be published at a later date. The study is expected to be completed by December 2021.

**Conclusions:**

Results of this study will be used to inform integration of the Kidney Score Platform within primary care settings so that it can serve as a central component of the National Kidney Foundation public awareness campaign to educate, engage, and empower individuals at risk for and living with chronic kidney disease.

**International Registered Report Identifier (IRRID):**

PRR1-10.2196/22024

## Introduction

Chronic kidney disease affects 37 million Americans [1]. Chronic kidney disease progression can ultimately lead to kidney failure, a life-threatening illness that, even with dialysis treatment, confers a death rate worse than most cancers and significantly reduces quality of life [2]. Additionally, chronic kidney disease is a disease multiplier that often occurs with diabetes or hypertension and increases the risk of emergency department visits, hospitalizations, and cardiovascular events [3]. Total Medicare spending for beneficiaries with chronic kidney disease and kidney failure was over $120 billion in 2017, of which over $35.9 billion was spent to manage or treat kidney failure, a condition that can often be prevented with optimal chronic kidney disease management [2].

Chronic kidney disease is usually asymptomatic. Optimal management slows the progression of kidney disease and reduces cardiovascular events [4,5]. Individuals cannot readily know their disease status or risk without clinician recognition of risk, testing, detection, and communication [6]. Previous studies [7,8] show that clinician detection and communication about kidney disease are suboptimal. Importantly, clinician diagnosis of chronic kidney disease is associated with increased delivery of evidence-based care, as well as increased patient awareness of their kidney disease [9-11]. As many as half of patients with advanced chronic kidney disease are unaware that they have chronic kidney disease, including those with laboratory manifestations of their kidney disease [12]. Patient awareness of chronic kidney disease, including the knowledge of having a kidney problem and the perceived risk of developing kidney disease, as well as the ability to affect their kidney health, is necessary for patients to participate in shared decision making about their kidney health and to apply management recommendations to improve outcomes [13]. Existing education programs, awareness campaigns, and clinical practice guidelines, including those from the National Kidney Foundation’s (NKF) Kidney Disease Outcomes Quality Initiative and the Veterans Affairs and Department of Defense Clinical Practice Guidelines for the Management of Chronic Kidney Disease, have minimally improved chronic kidney disease awareness in the United States and veteran populations, respectively [14,15].

Among the population served by Veterans Affairs (VA), 1 in 6 has chronic kidney disease. It is the fourth most diagnosed disease within the Department of Veteran Affairs, and over 13,000 veterans develop end-stage renal disease each year [16]. Diagnosis within the VA is low, with only 39% of veterans with chronic kidney disease stages 3-4 appropriately identified with International Classification of Diseases diagnostic codes in 2011 [7]. A roundtable discussion during the Kidney Innovation Summit hosted by the VA Center for Innovation and the American Society of Nephrology highlighted the need for a paradigm shift in education and awareness by addressing gaps in communication between “what is said, what is heard, and what the patient understands” and educating clinicians on how to meaningfully engage with patients at different stages of kidney health. This investigation is a significant incremental contribution to the Advancing American Kidney Health Initiative’s aim 1 of 3 to reduce the number of Americans developing end-stage renal disease by 25% by 2030 [17].

Implementation science frameworks, which take into account multiple interacting domains and processes that factor into successful program implementation, can help assure that the aforementioned gaps in chronic kidney disease awareness and education are addressed with interventions that are feasible and generalizable in real-world setting [18]. Consistent with these principles, the National Kidney Foundation developed the Kidney Score Platform to increase individual awareness and perceived risk of chronic kidney disease, thereby enhancing discussions about kidney disease between patients and clinicians. With this paper, we describe how we leveraged an implementation science framework to develop the Kidney Score Platform and propose to implement and evaluate its impact on veteran understanding and engagement with chronic kidney disease care as well as clinician self-efficacy for identifying and discussing chronic kidney disease with their patients. 

## Methods

### Behavior Change Wheel

**Figure 1:**
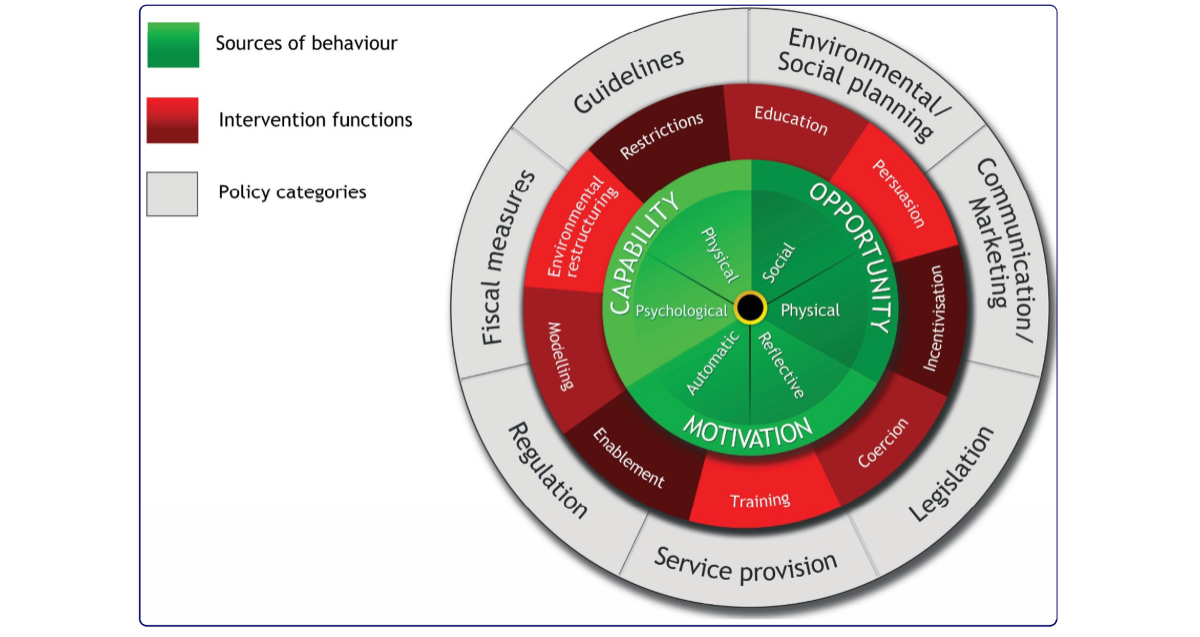
The behavior change wheel from reference Michie and colleagues [19].

We selected the Behavior Change Wheel [19] as a framework for the development of the Kidney Score Platform. This framework was developed from a synthesis of 19 other frameworks of behavior change and incorporates 3 layers to guide intervention development and deployment (Figure 1) [19]. The first layer uses the Capability, Opportunity, Motivation, Behavior (COM-B) model to identify barriers, enablers, and sources of behaviors that serve as potential intervention targets. The second layer of the wheel identifies 9 intervention functions that could be leveraged by the Kidney Score Platform to address the enablers and barriers to behavior change identified in the first layer. The third layer offers 7 policy options that can be deployed to support the testing and subsequent use of the Kidney Score Platform.

### The Kidney Score Platform

From June 2016 to September 2016, the NKF conducted in-depth interviews with 20 nondialysis-requiring chronic kidney disease patients and 11 clinician experts in chronic kidney disease care including nephrologists and primary care practitioners (Multimedia Appendix 1). These interviews identified information that patients found most useful at various stages of their chronic kidney disease journey and provided insight into the type of information that clinicians conveyed to patients at those stages. Data from both clinician and patient audiences also identified barriers and enablers to behavior change that were categorized according to the COM-B model. Examples of patient barriers included the asymptomatic nature of disease, reliance on primary care clinicians to determine risk and order testing, and inadequate kidney-focused clinical workforce (ie, primary care clinicians, chronic kidney disease certified nutritionists, and nephrologists) (capability); underdiagnosis of chronic kidney disease in primary care and limited public awareness of chronic kidney disease (opportunity); and limited kidney literacy and low patient activation (motivation). Patient enablers included access to patient portals containing laboratory findings, availability of chronic kidney disease care planning, and care coordination (capability); availability of online chronic kidney disease patient education and access to chronic kidney disease care management tools (opportunity); and participation in chronic kidney disease peer mentoring programs and availability of self-management programs for chronic kidney disease (motivation).

Examples of clinician barriers were limited awareness of chronic kidney disease clinical practice guidelines, challenges in modifying organizational workflows for diabetes and hypertension, no financial incentives for chronic kidney disease recognition (capability); limited availability of chronic kidney disease training in medical school and absence of formal chronic kidney disease awareness campaigns (opportunity); low prioritization of chronic kidney disease by health care organizations and by health care payers; and limited data analytics to highlight chronic kidney disease care gaps (motivation). Clinician enablers included chronic kidney disease clinical decision support in the electronic health record, performance measures for chronic kidney disease testing, and risk adjustment strategies for chronic kidney disease diagnosis and severity (capability); systemic quality improvement focused on chronic kidney disease care processes and inclusion of chronic kidney disease interventions in risk factor education (ie, hypertension) (opportunity); and use of practice facilitation teams to improve chronic kidney disease care and use of registries to demonstrate opportunities in chronic kidney disease care (motivation).

### Kidney Score Platform Online Interface (for Patients)

Based on the aforementioned behavior change targets, the NKF’s multidisciplinary team of experts in web technology design, user interface development, information architecture, adult learning, and patient education partnered with experts in kidney care, patient education, and app design to develop the Kidney Score Platform online interface. Using the Behavior Change Wheel intervention functions education, persuasion, and enablement, the interface was designed to improve awareness and understanding about kidney disease among people at risk for and living with chronic kidney disease (Table 1).

**Table 1:** Elements of the Kidney Score Platform informed by the 3 layers of the Behavior Change Wheel framework to directly address patient and clinician barriers and facilitators of behavior change for chronic kidney disease awareness, communication, and management.

Elements		Behavior change			Behaviour Change Wheel		
		Barriers	Facilitators	Behavior sources		Intervention functions	Policy categories
**Patient-facing**							
	**Risk online interface**						
		Asymptomatic nature	Patient portals with laboratory data	Capability		Education	Communication/ marketing
		Limited public awareness	Online education	Opportunity		Education	Communication/ marketing
		Underdiagnosis	Access to management tools	Opportunity		Persuasion	Regulation
		Limited health literacy; low patient activation	Existing self-management programs	Motivation		Enablement	Regulation
**Clinician-facing**							
	Practice assessment	Suboptimal awareness of clinical practice guidelines	Quality improvement focused on chronic kidney disease	Opportunity		Persuasion	Regulation; communication/ marketing
	CKDinform 2.0	Absence of formal awareness campaigns	Linkage of chronic kidney disease with risk factor education	Opportunity		Education	Guidelines
	AHRQ REALM-SF^a^	Poor knowledge about health literacy	Validated tools to quickly assess health literacy	Motivation		Education	Regulation
	Teach-back video	Limited training in medical school	Linkage of chronic kidney disease with risk factor education	Capability		Modeling	Communication/ marketing; Regulation
	Change Package	Challenges in modifying workflows for diabetes and hypertension	Clinical decision support in the electronic health record	Capability		Enablement	Service provision

^a^AHRQ REALM-SF: Agency for Healthcare Research and Quality Rapid Estimate of Adult Literacy in Medicine—Short Form.

The 3 other policy categories described in the Behavior Change Wheel that will not be applied to the evaluate the Kidney Score Platform are fiscal, legislation, and social planning.

**Figure 2:**
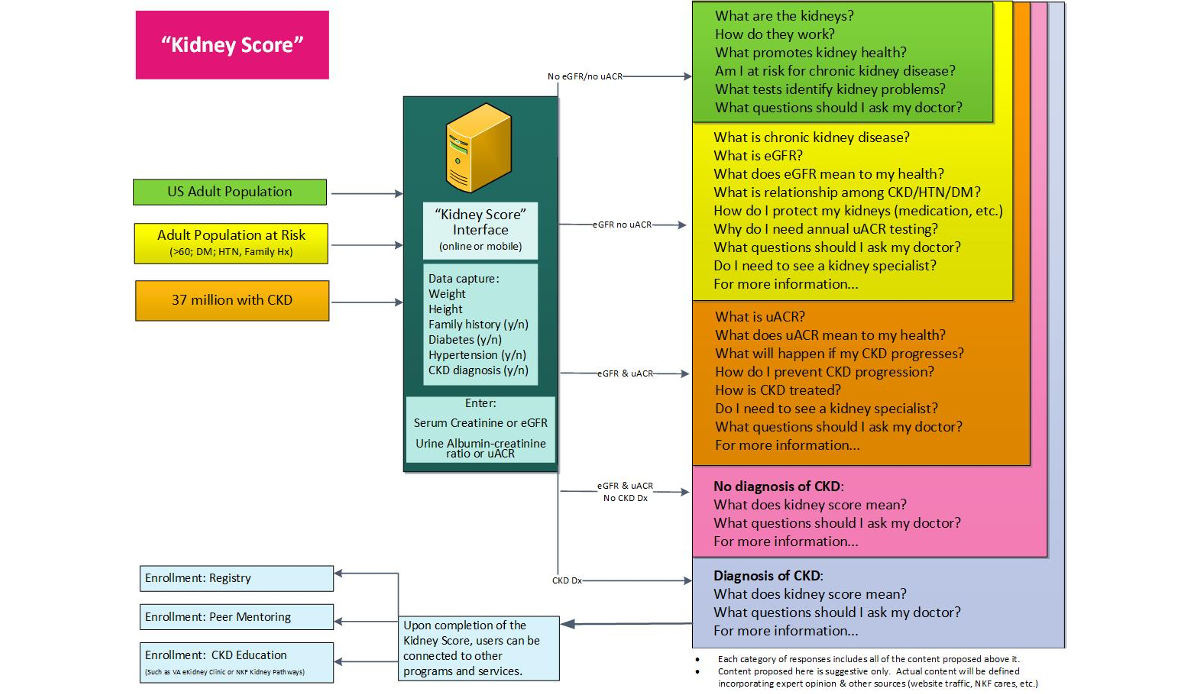
Design of the Kidney Score Platform online interface, which utilizes a rule engine and risk predictive analytics to provide interactive health information tailored to the individual’s chronic kidney disease risk and health status. CKD: chronic kidney disease; eGFR: estimated glomerular filtration rate; DM: diabetes; HTN: hypertension; uACR: urine albumin-creatinine ratio.

The free online interface utilizes a rule engine and risk predictive analytics to provide interactive health information tailored to the individual’s chronic kidney disease risk and health status. After entering risk factor information as well as laboratory findings in the Kidney Score Platform’s online interface, end users receive educational programming tailored to their current clinical status and risk for chronic kidney disease development or progression. Information is organized into context-specific easily digestible snippets, an approach aligned with the growing body of health education on how interactive content and tools such as apps, online health assessments, calculators, games, and quizzes can significantly affect comprehension, attitudes, self-efficacy, and health-related behavior change (Figure 2) [20-22]. The Kidney Score Platform Online interface blends the two of the most frequently accessed areas on the NKF website [23] (which receive over 19 million unique visitors per year)—the chronic kidney disease Risk Stratification Tool (Heat Map) and the patient-directed A to Z Guide—into a single tailored learning experience about kidney health. Topics span the spectrum of information from risk factors for chronic kidney disease to the benefit of medical nutrition therapy, fitness, weight control, and informed decision making for medical management of chronic kidney disease.

### Clinical Practice Toolkit (for Clinician Teams)

Clinician-identified barriers and enablers for optimal discussions about chronic kidney disease informed the creation of the Kidney Score Platform’s Clinical Practice Toolkit. Leveraging the Behavior Change Wheel intervention functions of education, persuasion, enablement, and modeling, the toolkit assists clinicians in discussing chronic kidney disease with individuals at risk of and those living with chronic kidney disease who may have different health literacy levels (Table 1). The toolkit includes elements that help identify patient populations at risk for and with chronic kidney disease, online continuing medical education about kidney disease detection and management, a tool that can be applied in clinical practice to assess patient health literacy for education tailoring, teach-back videos that model discussions about chronic kidney disease concepts, and quality improvement interventions that can be implemented at the clinic level to improve identification of patients at risk or living with chronic kidney disease.

*Practice assessment* is an algorithm that extracts chronic kidney disease-related data from the electronic medical record to assist clinical practices in identifying (1) patients at risk for chronic kidney disease who have not been tested for kidney disease; (2) patients with underlying undiagnosed chronic kidney disease based on laboratory testing; (3) patients with chronic kidney disease who could benefit from improved understanding of chronic kidney disease and the choices for medical management of chronic kidney disease.

*CKDinform 2.0* is an online continuing education activity based on Kidney Disease Outcomes Quality Initiative clinical practice guidelines that review chronic kidney disease testing, detection, and care [24].

The *Rapid Estimate of Adult Literacy in Medicine—Short Form* (REALM-SF) is a validated 7-item word recognition test that provides clinicians with a quick assessment of a patient’s health literacy and capacity to understand health information [25].

*Teach-back videos* provide examples for practitioners of how to utilize the Kidney Score Platform online tool to communicate with individuals at risk for or living with chronic kidney disease. These short videos, featuring an expert clinician investigator and an individual living with chronic kidney disease, provide several examples of the teach-back method being utilized to assess patient comprehension of chronic kidney disease–related concepts (ie, understanding the importance of kidney health, medical nutrition therapy for chronic kidney disease, and importance of avoiding certain over-the-counter medications) including instructions conveyed during the office visit about medication safety use.

*Change Package* is a free publicly available online compendium of tools or actionable process improvements that clinicians in primary care settings can implement to integrate Kidney Score Platform chronic kidney disease education and related interventions into their practice workflow [26]. By including process improvements that can be rapidly tested, the Change Package can help practices to deploy systems that efficiently and effectively support patients with chronic kidney disease. It was developed in collaboration with the Centers for Disease Control and Prevention Million Hearts initiative based on the foundation of their Hypertension Change Package [27].

### Study Design

The policy strategies from the Behavior Change Wheel that enable and support the testing and use of the Kidney Score Platform include communication/marketing, regulation, guidelines, and service provision (Table 1). These are embedded in the pre–post study design that will test the impact of the Kidney Score Platform on patient self-efficacy for chronic kidney disease management within the primary health care settings of 2 VA medical centers: VA New York Harbor Healthcare System and VA Connecticut Healthcare System. This study has been approved by the Institutional Review Boards of the VA Connecticut Healthcare System (#02290) and VA New York Harbor Healthcare System (#01705) and any modifications to the study protocol will be communicated to them for review before implementation.

### Intervention

**Figure 3:**
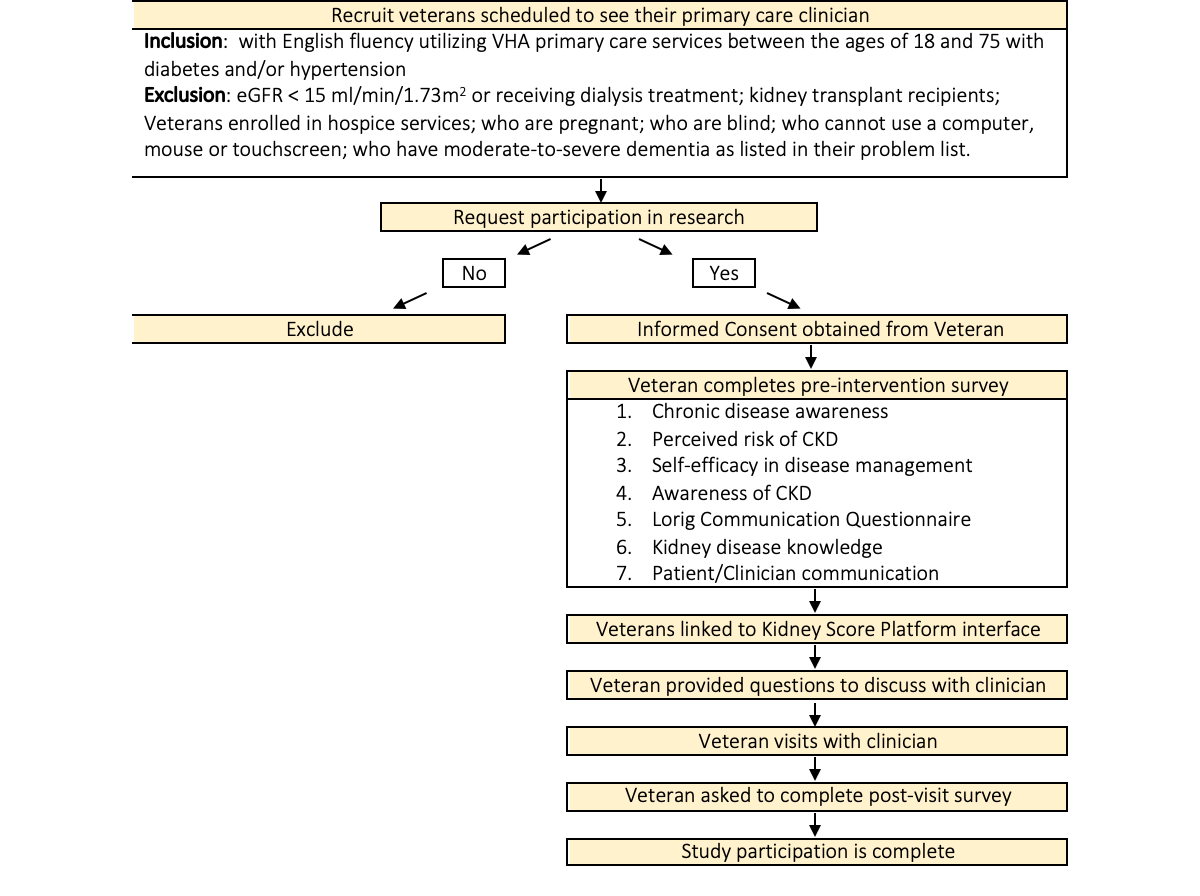
Process flow for Kidney Score study. CKD: Chronic Kidney Disease; eGFR: estimated glomerular filtration rate; VHA: Veteran's Health Administration.

Veterans will interact with the Kidney Score Platform online interface individually while awaiting their clinical encounter via tablets or laptops (communication/marketing). As depicted in Figure 3, veterans who have consented to participate in the study will complete an online preassessment survey before exposure to the Kidney Score Platform’s Online Interface. Participants will then engage with the online interface for 5-20 minutes before their clinician visit. This interaction can occur at home for a telehealth visit or in the waiting room for an in-person visit. If requested, Kidney Score Platform will offer participants a copy of questions to ask their clinician about their kidney health, tailored to their chronic kidney disease risk, determined by the Kidney Score Platform’s embedded rules engine. These questions aim to further reinforce important chronic kidney disease topics and foster meaningful patient-professional conversations (regulation). Within a week after the clinical encounter, veterans will be asked to complete a brief survey (electronically or by phone) to assess the impact of the Kidney Score Platform on their understanding of chronic kidney disease and the quality of their conversation with their practitioner about kidney health. The Kidney Score Platform is intended to serve as an adjunct to clinical care; there will be no restrictions on concomitant care interventions delivered during or after the clinical visit.

Prior to veteran recruitment, we will offer the accompanying clinical practice toolkit to primary care teams (service provision). Practitioners will be encouraged (but not mandated) to utilize the clinical practice tools, particularly completing CKDinform 2.0 and watching the chronic kidney disease teach-back video (communication/marketing). These tools will prepare practitioners for conversations with veterans as well as reinforce chronic kidney disease clinical practice guidelines on detection, patient education, and intervention (regulation, guidelines). Pre and postsurveys will be conducted to assess the impact of the clinical practice toolkit on practitioners’ perception of chronic kidney disease, chronic kidney disease knowledge, and important topics for chronic kidney disease patient education.

### Study Population Criteria

Veterans with and at risk for chronic kidney disease and their primary care clinical teams represent the target population. English-speaking veterans utilizing VA primary care services between the ages of 18 and 75 with diabetes or hypertension as defined in the electronic health record will be eligible for this study. Exclusion criteria include an estimated glomerular filtration rate <15 mL/min/1.73m^2^ from recent laboratory tests in the medical record or those receiving dialysis treatment, as the Kidney Score Platform is not geared toward individuals with severe chronic kidney disease. Kidney transplant recipients will also be excluded, as their care is delivered predominantly in the subspecialty care setting rather than the primary care setting. We will also exclude veterans enrolled in hospice services, as chronic kidney disease awareness and self-management are less important for this population. Other exclusions include pregnancy, vision impairment, and severe dementia, identified by the problem list in the electronic health record or during the consent process. All primary care clinicians working as part of a Patient Aligned Care Team (PACT) in participating VA Medical Centers will be eligible for participation in the study.

### Recruitment

Veterans work collaboratively with their PACT multidisciplinary team members to meet their health care needs. PACTs often include clinicians, nurses, medical assistants, social workers, and pharmacists, among other allied health professionals. The study team will work with each PACT team’s care coordination staff to identify veterans who meet the inclusion criteria with appointments during the intervention’s phase. Collaborating with primary care champions at each site, the project team will explore variations in recruitment process based on the site’s resources, clinical flow, and use of telehealth for care delivery due to the COVID-19 pandemic.

Recruitment of clinicians from each PACT team will occur during a presentation of the project, during which the research team can answer questions. Clinical champions from each participating VA have been engaged in project co-design and will help ensure adequate and diverse clinician participation.

### Consent

A member of the project team will describe the innovation and its goals and purpose to eligible veterans. Individuals that can correctly explain the project protocol and answer simple teach-back questions (in part to assess for dementia) will be asked to provide consent. Eligible providers will also be asked to provide consent to participate after they hear about the project and its goals.

### Data Collection and Outcomes

#### Sociodemographic Data

Sociodemographic data (age, gender, race/ethnicity), co-morbid conditions (diabetes, hypertension, cardiovascular disease), and baseline laboratory data (serum creatinine, proteinuria, or albuminuria), will be collected from all consenting participants from the electronic health record, including participants who do not complete surveys for primary outcome collection (see below). All individually identifiable data will remain within the VA data system. Practitioner sociodemographic data (age, gender, race/ethnicity), role, panel size or practitioner caseload, and years of experience will be self-reported.

#### Behavior and Attitude Outcomes

The primary outcome is change in self-efficacy for chronic kidney disease management, measured by the Patient Activation Measure [28]. We will also examine changes in self-reported communication with practitioners about chronic kidney disease and ability of veterans to describe the tests used to detect chronic kidney disease. Validated instruments will be used [29,30], deployed via secure online surveys linked to the Kidney Score Platform’s online interface to maximize data quality and security. We will also assess the impact of the Clinical Practice Toolkit on practitioners’ perception of chronic kidney disease, chronic kidney disease knowledge, and important topics for chronic kidney disease patient education (Table 2).

**Table 2:** Patient and clinician behavior outcomes.

Outcome	Method of ascertainment
Change in patient self-efficacy for disease self-management	Patient Activation Measure [28]
Change in patient perceived risk of chronic kidney disease and chronic kidney disease awareness	Boulware questionnaire [31]
Change in patient comfort communicating with providers	Stanford communication instrument [29]
Change in clinician perception of and actual chronic kidney disease knowledge	Knowledge survey

#### Process Measures

Using the Reach Evaluation-Adoption Implementation and Maintenance (RE-AIM) framework for program evaluation [32], we will conduct process evaluation of the Kidney Score’s online platform and accompanying Clinical Practice Toolkit, across and within VA partnering sites. We will examine (1) reach to intended target audiences (veterans most at risk for chronic kidney disease, PACT team members); (2) effectiveness of the Kidney Score among veterans and practitioners, including usability of the online interface; (3) adoption of the Kidney Score and toolkit by primary care teams across and within partnering VA Medical Centers and (4) implementation consistency, costs, and adaptations made during deployment and delivery (Table 3).

**Table 3:** Process outcomes for implementation analysis, based on the RE-AIM framework [32].

RE-AIM^a^ dimension	Outcome	Method of assessment
Reach (representativeness of participants)	1. Number of eligible patients who consent	VAMC^b^ clinic lists; electronic health record data; description of PACT^c^ teams
	2. Demographic and clinical characteristics of subjects who enroll/do not enroll	
	3. Demographic characteristics of participating clinicians	
Effectiveness (adherence and engagement)	1. Usability of the Kidney Score Platform online interface	Screen sharing and observation
	2. Percentage of patients who request a copy of discussion questions for their provider	
	3. Percentage of at-risk patients with urine albumin-creatinine ratio testing	Electronic health record data
	4. Percentage of patients with chronic kidney disease with diagnosis on problem list	
	5. Percentage of patients with proteinuria prescribed angiotensin converting enzyme inhibitor/angiotensin receptor blocker	
	6. Percentage of patients referred to nephrology	
Adoption	1. Are providers satisfied with integration with clinic work flow?	Formative evaluation with PACT team members and Quality Improvement team leaders
Implementation	1. Is each component delivered as intended? (fidelity)	
	2. What components of the intervention were customized to each VAMC?	
	3. How much VAMC personnel time is required to deploy the clinical practice toolkit?	

^a^RE-AIM: Reach Evaluation-Adoption Implementation and Maintenance.

^b^VAMC: Veteran Affairs Medical Center.

^c^PACT: Patient Aligned Care Team.

### Sample Size Considerations

The study is powered to detect changes in Patient Activation Measure (PAM) score, a validated measure of an individual’s knowledge, skill, and confidence in managing one’s health [28]. Data from prior self-management intervention studies have demonstrated a change in PAM score SD of 14.5. Using two-tailed paired *t* test calculations, we would need to enroll 103 veterans to engage with the Kidney Score Platform and complete the pre and postsurveys to detect a 4-point clinically meaningful change in the PAM, assuming that the PAM would not change among individuals not exposed to Kidney Score Platform [33]. Accounting for a conservative 20% dropout, we need to recruit 124 veterans. In previous studies [30] related to chronic kidney disease education in primary care, 67% of eligible patients agreed to participate. Preliminary data suggest that there are >1200 eligible veterans for our project in each medical center PACT program.

### Data Analysis

Impact of the Kidney Score Platform on change in participant PAM scores (primary outcome) will be conducted with linear mixed models, adjusting for age, gender, diabetes status, kidney disease severity, and clinic site. Additional analyses will look for effect modification by clinician knowledge of chronic kidney disease. Similar methods will be used to assess secondary outcomes: self-reported communication with practitioners about chronic kidney disease and ability of veterans to describe the tests used to detect chronic kidney disease.

## Results

As of September 2020, usability studies are underway with veterans and clinicians to refine the patient-facing components of the Kidney Score Platform before study initiation. Results and subsequent changes to the Kidney Score Platform will be published at a later date. The study is expected to be completed by December 2021.

## Discussion

This paper illustrates a theory-informed and evidence-based approach to developing and testing an intervention to enhance communication about kidney disease among patients and health care professionals. To develop all of the elements of the Kidney Score Platform, we leveraged the Behavior Change Wheel framework to directly target behaviors that were identified as barriers to optimal chronic kidney disease awareness and communication during preparatory interviews, using evidence-based intervention functions [19]. To test The Kidney Score Platform’s impact, we partnered with VA leaders to identify the policy categories from the Behavior Change Wheel that could be employed at the organizational level to support its implementation. We are now poised to test the Kidney Score Platform’s efficacy and effectiveness on increasing an individual’s self-efficacy and activation to decrease chronic kidney disease risk and a primary care team’s ability to manage chronic kidney disease and communicate about kidney disease.

While several chronic kidney disease education programs and awareness campaigns exist across the United States, there are limited examples in the literature of how theoretical frameworks can be leveraged to develop and test interventions to enhance kidney-related communication among clinicians and patients and improve self-efficacy and overall health among individuals with kidney disease. To our knowledge, this is one of the few chronic kidney disease awareness interventions developed and proposed to be tested according to an individual behavioral change theory (ie, Behavior Change Wheel). Theory-informed interventions are more likely to be effective than interventions not based on theory, though a formal study is required to test whether the slow deliberate process (such as that used to develop the Kidney Score Platform) leads to greater effectiveness within the context of health care delivery.

Results of the anticipated study examining the impact of Kidney Score Platform on patients and clinician chronic kidney disease awareness and communication will be shared with participants and will used to refine the different elements of the platform and their integration within primary care settings including new care delivery workflows, such as telehealth. Study results will be most applicable to care delivery in VA administration settings involving in-person and phone or video visits. While this is one limitation to this study, we anticipate that the Kidney Score Platform’s impact on awareness, self-efficacy, educational, and communication outcomes among veterans and their clinicians will be generalizable to other settings and novel care delivery mechanisms. If so, the robust process by which the Kidney Score Platform was developed can serve as a model for the creation and implementation of other innovations that focus on behavior change as a means of enhancing kidney health. The refined Kidney Score Platform that will emerge from this study has the potential to significantly impact the lives of the approximately 37 million US adults affected by chronic kidney disease, as it will serve as a central component of the NKF’s national public awareness initiative to educate, engage, and empower individuals at risk for and living with chronic kidney disease.

## Appendix

Multimedia Appendix 1 Kidney Score Platform design interview probe.

## Abbreviations

COM-B: capability, opportunity, motiviation – behavior
NKF: National Kidney Foundation
PACT: Patient Aligned Care Teams
PAM: Patient Activation Measure
RE-AIM: Reach Evaluation-Adoption Implementation and Maintenance
VA: Veterans Affairs
