# Supplementary material for: The Kidney Score Platform for Patient and Clinician Awareness, Communication, and Management of Kidney Disease: Protocol for a Mixed Methods Study
Source: JMIR Res Protoc. 2020 Oct 19;9(10):e22024. doi: 10.2196/22024 (PMC7605977; doi:10.2196/22024)
Supplement: Multimedia Appendix 1 [file resprot_v9i10e22024_app1.docx]

# CKD Qualitative Patient Interviews

Understanding the CKD Patient Journey and Information Needs

I am XXXXXXXX at the National Kidney Foundation. On behalf of NKF, I would like to thank you for agreeing to speak with us regarding your experience living with a chronic kidney disease. We appreciate your support of our mission very much.

For the purposes of this discussion, I will be using the term “CKD” to encompass diagnosis of chronic kidney disease.

This interview is being recorded and there at least two other members of the NKF team transcribing this discussion as it occurs. In order to ensure your privacy, we ask that you do not mention any identifying information, such as your name, city or even the name of the physician that provides your care. To ensure your privacy, I will not be using your last name during this interview.

This discussion will require about an hour. We need your full attention during this time. As we move through this discussion, I will provide you with information regarding our progress and the time remaining for the discussion.

So let’s begin…

1. Has your doctor told you that you have CKD?
2. What type of doctor provided you that diagnosis?

*[Probe on the specialty of the physician who provided the diagnosis]*

- 1. Are you still under the care of this doctor?
  2. Do you see more than one physician for your CKD care?

*[Probe for specialty of additional physicians on care team]*

- 1. Are there other healthcare professionals, such as nurse practitioners or physicians assistants that provide CKD-related care?

1. When were you diagnosed with CKD?
2. Do you have diabetes or hypertension? When were you diagnosed for those conditions?
   1. What medications are you on to treat these conditions?
3. What events triggered your diagnosis of CKD?
4. At what stage of CKD were you diagnosed?

1. When you received your diagnosis of CKD, how did your physician explain CKD to you?
2. When you were first diagnosed, did your physician provide you with suggestions regarding lifestyle changes you should make? What were they?

*******

1. Do you interact with other people who may have CKD?
   1. If yes,
      1. How did you meet them?
      2. How often to you speak/communicate with them?
      3. Does this interaction help you? How?
         1. If no,
            1. Why not?
2. Are you a member of any CKD-related organizations?
   1. Are you aware of the services that these organizations offer to patients like yourself?

*[Probe: If aware, which of their services do you use?]*

1. Do you use the internet to obtain information regarding CKD?
   1. If so, what websites do you visit?
   2. Are any sites particularly useful?
2. Are there any internet sites – that are not CKD specific – that you are aware of that offer interesting services or support - that you wish were available for CKD support?
3. As a person living with CD, can you think of or describe any services or support that would be particularly useful for you? If you could create a wish list of what support would be helpful to you as a CD patient to learn about your disease and treatment, and to live with the disease, what would be on your list?
4. As a person living with CKD, is there anything that you wish your physician had done differently regarding your care or education?

# NKF Qualitative Clinician Interviews

**Kidney Score Platform Logic Model Development - July 2016**

The goal of this conversation is to understand how clinicians provide information to their patients across the spectrum of CKD. We are simply looking for your top of mind response to the patient vignettes we have outlined below. There is no incorrect answer or approach – our goal is to simply record the strategy that you would employ in speaking with the patients described.

I'm going to walk you through a conversation you might have with three different patients. We would just like to hear what you might say to them to explain CKD to them.

We would like to consider each separate patient vignette and tell us what you would say to each patient about CKD assuming this is your first conversation with them about CKD.

**Patient 1**

The first patient is a 55-year-old man with diabetes. His annual blood work has come back with a GFR of 45. What information would you provide him about CKD in your first encounter with him?

Probe: Is there any additional information you would provide?

Are there materials you might distribute?

**Patient 2**

The next patient female, she's 60 years old and she has both hypertension and diabetes. Her most recent GFR is 29. As a newly diagnosed patient what would you tell her?

Probe: Is there any additional information you would provide?

Are there materials you might distribute?

**Patient 3**

The last patient is a 64-year-old man with diagnosed chronic kidney disease. His GFR is at 18 and what information would you provide him with at this point in his progression?

Probe: Is there any additional information you would provide?

Are there materials you might distribute?

We have one last question. We are trying to determine the most effective way to translate the complex idea of declining kidney function to patients in the most descriptive way possible. We are trying to identify metaphors that can be used to assist with this. For instance, some people describe having a heart attack as feeling like there is an elephant sitting on their chest.

Are there metaphors that you utilize to describe CKD to your patients?

This concludes our interview. We’d like to thank you again for taking the time to speak with us and for your ongoing support of the National Kidney Foundation.
